# Early fluid balance and mortality following extracorporeal cardiopulmonary resuscitation: a high volume, single center study

**DOI:** 10.1186/s13049-025-01381-8

**Published:** 2025-04-22

**Authors:** Humphrey GM Walker, Alexander S Richardson, Arne Diehl, Brooke Riley, Eldho Paul, Aidan Burrell

**Affiliations:** 1 Department of Intensive Care and Hyperbaric Medicine, The Alfred, Melbourne, VIC Australia; 2 Department of Critical Care, St Vincent’s Hospital, 41 Victoria Parade, Fitzroy, Melbourne, VIC 3065 Australia; 3 Department of Critical Care, University of Melbourne, Melbourne, VIC Australia; 4 Australian and New Zealand Intensive Care Research Centre, School of Public Health and Preventive Medicine, Monash University, Melbourne, VIC Australia

**Keywords:** ECPR, ECMO, Fluid, Cardiac arrest

## Abstract

**Background:**

For patients supported with venoarterial extracorporeal membrane oxygenation (VA-ECMO), a positive cumulative fluid balance at day three has been associated with increased mortality. However, there is limited evidence examining this association in patients requiring extracorporeal cardiopulmonary resuscitation (ECPR). The aims of this study were to (1) to describe contemporary fluid practice in patients requiring ECPR and (2) assess the relationship between early cumulative fluid balance and 28-day mortality.

**Methods:**

This was a retrospective, single centre, observational study using data collected from the EXCEL registry and the hospital electronic medical record. All patients undergoing ECPR from January 2017 until December 2022 were identified using a prospectively collected database. Patients aged < 18 years old or had extra-corporeal support ceased prior to arrival to the intensive care unit were excluded. Fluid data was collected for days 1,2,3 and 7; and cumulative balances reported for day 3 and day 7.

**Results:**

104 patients were identified, of which 100 were included. The mean age was 48.9 (SD 14.1) years, 72 (72%) were male. 54 (54%) were out-of-hospital cardiac arrests. Median low flow time was 43 (IQR 39–76) minutes. 51 (51%) had died by day 28. After adjusting for location of cardiac arrest, return of spontaneous circulation and duration of ECMO, a 1 L increase in cumulative fluid balance to the end of day 3 was not independently associated with 28-day mortality (adjusted OR 1.09 [95% CI 0.97–1.22]), however by day 7 this was independently associated with an 11% increased risk of 28-day mortality (adjusted OR 1.11 [95% CI 1.001–1.23]).

**Conclusion:**

A one litre increase in CFB at the end of day 3 was not associated with 28-day mortality; but a one litre increase in CFB by the end of day 7 was associated with an 11% increase in the odds of day 28 mortality. The impact of restrictive fluid management strategies in those requiring ECPR should be assessed in prospective trials.

**Supplementary Information:**

The online version contains supplementary material available at 10.1186/s13049-025-01381-8.

## Background

Fluid administration in intensive care unit (ICU) patients is common [1] and not without harm [2]. In the general ICU population, adjusted mortality risk may increase by 20% per additional litre increase in fluid balance [3]. In patients supported by extracorporeal membrane oxygenation (ECMO), the optimal fluid regimen is uncertain. Fluid overload is common [4]: fluid leak, bleeding, and low ECMO blood flow all can necessitate fluid resuscitation leading to positive fluid balances. Negative fluid balances, however, have been associated with improved survival for both patients on venovenous (VV)-ECMO [5] and venoarterial (VA)-ECMO [6, 7]. There is limited literature that describes the type of fluid administered, or the impact of cumulative fluid balances (CFB) on outcomes in those requiring extracorporeal cardiopulmonary resuscitation (ECPR). Further work assessing both these facets is important to ascertain if fluid administration is a potentially clinically modifiable factor in ECPR patients. Accordingly, the aims of this study were to firstly describe the types of fluids commonly used during ECPR resuscitation and maintenance and secondly assess the relationship between cumulative fluid balance and mortality in patients who require ECPR.

## Methods

This was a single centre, retrospective, observational study conducted at the Alfred Hospital, Melbourne. The Alfred Hospital is a metropolitan, quaternary hospital in Melbourne, Australia with a 24/7 ECPR service. This includes both out-of-hospital cardiac arrests (OHCA) and in-hospital cardiac arrests (IHCA). Cannulation for OHCA could be pre-hospital as part of the CHEER-3 trial [8] or in the hospital emergency department for those transported with CPR ongoing. All patients receiving ECPR between 1st January 2017 and 31st December 2022 were included for analysis unless they met exclusion criteria. Exclusion criteria included paediatric (< 18 years) patients and those in whom ECMO support was withdrawn prior to ICU admission. Patients were identified using The Australian and New Zealand ECMO Registry (EXCEL), and an internal database of ECMO patients prior to EXCEL commencement. EXCEL is coordinated by the Australian and New Zealand Intensive Care Research Center (Monash University) and collects demographics, diagnostics, therapies, morbidity and mortality outcomes on patients requiring ECMO at major hospitals in Australia and New Zealand [9]. Local ethical approval was obtained prior to collection of any data (Alfred Health HREC 636/23).

### Data collection and definitions

Data was sourced from both local hospital electronic medical records and the EXCEL Registry. Data collected included the type and volume of fluid administered over the first 7 days post ECMO initiation alongside demographic data, cardiac arrest characteristics and outcome data. Fluid data was collected for days 1,2,3 and 7. Day 1 was defined as the first day of ECMO. Each day commenced at 0000 and ended at 2359. Total fluid input was defined as the sum of all fluids received for that period. This included any resuscitation, maintenance, drugs, and feeds. CFB was defined as total fluid input minus total fluid output. Fluid output included urine output, renal replacement therapy effluent, GI aspirates and drain output. The volume and type of fluid that was administered additional to that required for drug administration / dilution was recorded. This was termed additional fluid. These volumes were censored at death, decannulation or day 7. The primary outcome was day 28 mortality. Cumulative fluid balances were assessed daily to the end of day 3 and at day 7 [10]. A full list of definitions used are included in the supplementary appendix.

### Statistical analysis

Continuous variables were summarised using mean (SD) or median (IQR) according to data type and distribution. Categorical variables were presented as counts and percentages. Comparisons between groups (28-day mortality Yes vs. No) were made using Student’s t-test or Mann-Whitney U test for continuous variables and chi-square or Fisher’s exact test as appropriate for categorical variables. A multivariate logistic regression model was used to assess the impact of cumulative fluid balance at the end of day 3 and day 7 on 28-day mortality with results presented as odds ratios (OR) and 95% confidence intervals (95% CI). Baseline variables were considered for inclusion in the model if significant (p value < 0.05) on univariate analysis and deemed clinically relevant. A post-hoc sensitivity analysis excluding early deaths (< 48 h) was conducted.

The Kaplan-Meier product-limit method was used to plot 28-day mortality as a function of time and comparisons between cumulative fluid balance quartiles were made with the log rank test. Missing data was not imputed.

All calculated p-values were two-tailed and a p value < 0.05 was chose to indicate statistical significance. Analyses were performed using SAS version 9.4 (SAS Institute, Cary, NC, USA). GraphPad Prism (Version 10, Graph Pad, Boston, USA) was used to create figures.

## Results

Between 1st January 2017 and 31st December 2022 there were 17,642 ICU admissions. 104 patients required ECPR of which 100 were included in this study. Of the 4 excluded, 1 was aged under 18 years and 3 had ECMO ceased prior to arrival in ICU. Table 1 describes the patient demographics and cardiac arrest characteristics.

**Table 1:** Showing baseline characteristics of those who died by day 28 compared to those who survived to day 28

	Entire Cohort (*n* = 100)	Dead at day 28 (*n* = 51)	Alive at day 28 (*n* = 49)	*p* value
**Age**,** Years (mean**,** SD)**	48.9 (14.1)	48.3 (15.2)	49.5 (12.9)	0.68
**Male Sex (n**,** %)**	72 / 100 (72.0%)	36 / 51 (70.6%)	36 / 49 (73.5%)	0.75
**Weight**,** kg (mean**,** SD)**	81.1 (18.9)	85.4 (18.7)	77.1 (18.3)	0.04
**Pre-Existing Comorbidities**,** n (%)**				
MI	4 / 99 (4.0%)	3 / 50 (6.0%)	1 / 49 (2.0%)	0.62
Congestive Cardiac Failure	9/ 99 (9.1%)	3 / 50 (6.0%)	6 / 49 (12.2%)	0.47
PVD	2 / 99 (2.0%)	2 / 50 (4.0%)	0 / 49 (0.0%)	0.50
Connective Tissue Disease	5 / 99 (5.1%)	0 / 50 (0.0%)	5 / 49 (10.2%)	0.053
Diabetes Mellitus ^a^	14 / 99 (14.1%)	11 / 50 (22.0%)	3 / 49 (6.1%)	0.023
**Diagnosis**,** n (%)**				
AMI	47 / 100 (47.0%)	22 / 51 (43.1%)	25 / 49 (51.0%)	0.43
Myocarditis	3 / 100 (3.0%)	1 / 51 (2.0%)	2 / 49 (4.1%)	0.97
Primary Arrhythmia	14 / 100 (14.0%)	9 / 51 (17.6%)	5 / 49 (10.2%)	0.28
Acute Decompensated Heart Failure	3 / 100 (4.0%)	1 / 51 (2.0%)	2 / 49 (4.1%)	0.97
PE	9 / 100 (9.0%)	4 / 51 (7.8%)	5 / 49 (10.2%)	0.95
Toxic	3 / 100 (3.0%)	1 / 51 (2.0%)	2 / 49 (4.1%)	0.97
**Cardiac Arrest Characteristics**,** n (%)**				
OHCA	54 / 100 (54.0%)	33 / 51 (64.7%)	21 / 49 (42.9%)	0.03
Witnessed	81 / 99 (81.8%)	37 / 50 (74.0%)	44 / 49 (89.8%)	0.042
Bystander CPR	99 / 99 (100.0%)	50 / 50 (100.0%)	49 / 49 (100.0%)	1.00
Shockable Initial Rhythm	51 / 96 (53.1%)	26 / 51 (52%)	25 / 45 (55.6%)	0.73
Low Flow Time, minutes (Median, IQR)^b^	52.5 (39–76)	60 (40–80)	45 (37–73)	0.65
Any ROSC^c^	33 / 97 (34.0%)	11 / 49 (22.4%)	22/48 (45.8%)	0.015
**Biochemical parameters closest to ECMO cannulation**				
pH (mean, SD)	7.01 (0.29)	6.91 (0.28)	7.11 (0.27)	0.007
Bicarbonate, mmol/L (mean, SD)	16.2 (6.2)	15.1 (5.8)	17.2 (6.5)	0.19
Lactate, mmol/L (mean, SD)	9.8 (5.6)	10.7 (5.2)	9.01 (5.8)	0.22

*MI = myocardial infarction*,* PVD = peripheral vascular disease*,* AMI = acute myocardial infarction*,* PE = pulmonary embolus*,* OHCA = out-of-hospital cardiac arrest*,* ROSC = return of spontaneous circulation ECMO = extra corporeal membrane oxygenation*

^a^ This includes all types of diabetes mellitus

^b^ Low flow time is the time from commencement of chest compressions to ECMO cannulation

^c^ ROSC is assumed if there is documented evidence of ROSC at any point. This did not have to be sustained

The mean age was 48.9 (SD 14.1) years, and 72% (72/100) were male. 54% (54/100) had an OHCA. Median low flow time was 52.5 (IQR 39.0–76.5) minutes. 47% (47/100) had an acute myocardial infarction as a cause of their cardiac arrest. 34% (33/97) were documented to have at least one episode of return of spontaneous circulation (ROSC) prior to initiation of ECMO. 75% (75/100) of patients underwent ECMO cannulation at the bedside (as opposed to in the catheter laboratory, operating theatre, or pre-hospital). Increased rates of OHCA, lower rates of return of spontaneous circulation (ROSC) and a lower pH peri-cannulation were noted in 28-day non survivors.

**Table 2:** Showing ECMO, cardiovascular and ventilatory supports

	Entire Cohort (*n* = 100)	Dead at day 28 (*n* = 51)	Alive at Day 28 (*n* = 49)	*p* value
ECMO Parameters				
Location of Cannulation				
Bedside	75 / 100 (75.0%)	41 / 51 (80.4%)	34 / 49 (69.4%)	0.20
OT	5 / 100 (5.0%)	4 / 51 (7.8%)	1 / 49 (2.0%)	0.39
Cath Lab	12 / 100 (12.0%)	2 / 51 (3.9%)	10 / 49 (20.4%)	0.01
Pre-hospital	8 / 100 (8.0%)	4 / 51 (7.8%)	4 / 49 (8.2%)	1.00
**ECMO Blood flow**,** litres per minute (median**,** IQR)**				
Day 1 ^a^	3.2 (2.9–3.5)	3.3 (2.9–3.6)	3.1 (2.8–3.5)	0.43
Day 2	3.2 (3.0–3.6)	3.2 (2.9–3.6)	3.1 (3.0–3.5)	0.91
Day 3	3.2 (2.5–3.6)	3.5 (2.9–3.6)	3.0 (2.4–3.4)	0.06
**Angiography** ^**b**^	64 / 99 (64.6%)	29 / 51 (56.9%)	35 / 48 (72.9%)	0.10
**LV Decompression (n**,** %)**^**c**^	13 / 100 (13.0%)	6 / 51 (11.8%)	7 / 49 (14.3%)	0.71
**Ventilatory Parameters**,** mmHg (mean**,** SD)**				
Daily PaO_2_^d^	168 (110)	207 (143)	146 (78)	0.03
Daily CO_2_^d^	38 (5)	38 (7)	38 (4)	0.81
Delta CO_2_^e^	7.3 (16.8)	10.9 (20.7)	4.3 (12.1)	0.11
**High dose vasopressors**,** n (%)**^**f**^	58 / 95 (61.1%)	37 / 47 (78.7%)	21 / 48 (43.8%)	0.001
**CRRT on ECMO**,** n (%)**	63 / 98 (64.3%)	33 / 50 (66.0%)	30 / 48 (62.5%)	0.72

*ECMO = extra corporeal membrane oxygenation*,* OT = operating theatre*,* LV = left ventricular*,* CRRT = continuous renal replacement therapy*

^a^ ECMO flow on day 1 is ECMO flow rate at 4 h post initiation

^b^ Of those 64 undergoing angiography a culprit lesion was found in 46/64 (71.8%), with 23/29 and 23/35 finding a culprit lesion

^c^ LV decompression includes LV vent, Impella and IABP

^d^ Averaged over the first 3 days (readings taken at the same time as day 1)

^e^ Delta CO2 is the difference between PaCO2 prior to ECMO initiation to PaCO2 24 h later

^f^ High dose vasopressors are defined as a noradrenaline dose > 0.26mcg/kg/min or addition of vasopressin [11]

ECMO flow rates at on days 1–3 and requirement for left ventricular unloading were similar between groups. An increased number of non-survivors received high dose vasopressors. Additional supports are described in Table 2. Comparing 28-day non survivors with 28-day survivors, occurrence of bleeding was similar occurring in 33.3% (16/48) and 31.3% (15/48) respectively.

Table 3 summarises the outcomes for the patients included in this study. 51% (51/100) had died by day 28. Median ECMO duration was shorter in 28-day non survivors compared to 28-day survivors (2 vs. 6 days). 60% (60/100) survived ECMO decannulation, with 49% (49/100) and 41% (41/99) surviving to day 28 and day 180 respectively.

**Table 3:** Showing outcomes for patients included in the study

	Entire Cohort (*n* = 100)	Dead at day 28 (*n* = 51)	Alive at day 28 (*n* = 49)	*p* - value
**ECMO Duration**,** days (median [IQR])**	4.0 (2–8)	2.0 (1–4)	6.0 (4–10)	0.001
**ECMO survival**,** n (%)**	60 / 100 (60.0%)	11 / 51 (21.6%)	49 / 49 (100.0%)	0.001
**Hospital Survival**,** n (%)**	43 / 100 (43.0%)	0 / 51 (0.0%)	43 / 49 (87.8%)	-
**180-day survival**,** n (%)**	41 / 99 (41.4%)	-	41 / 48 (85.4%)	-

ECMO = extra-corporeal membrane oxygenation

Outcome data for 180-day survival was not available for one patient

### Fluid type

**Fig. 1:**
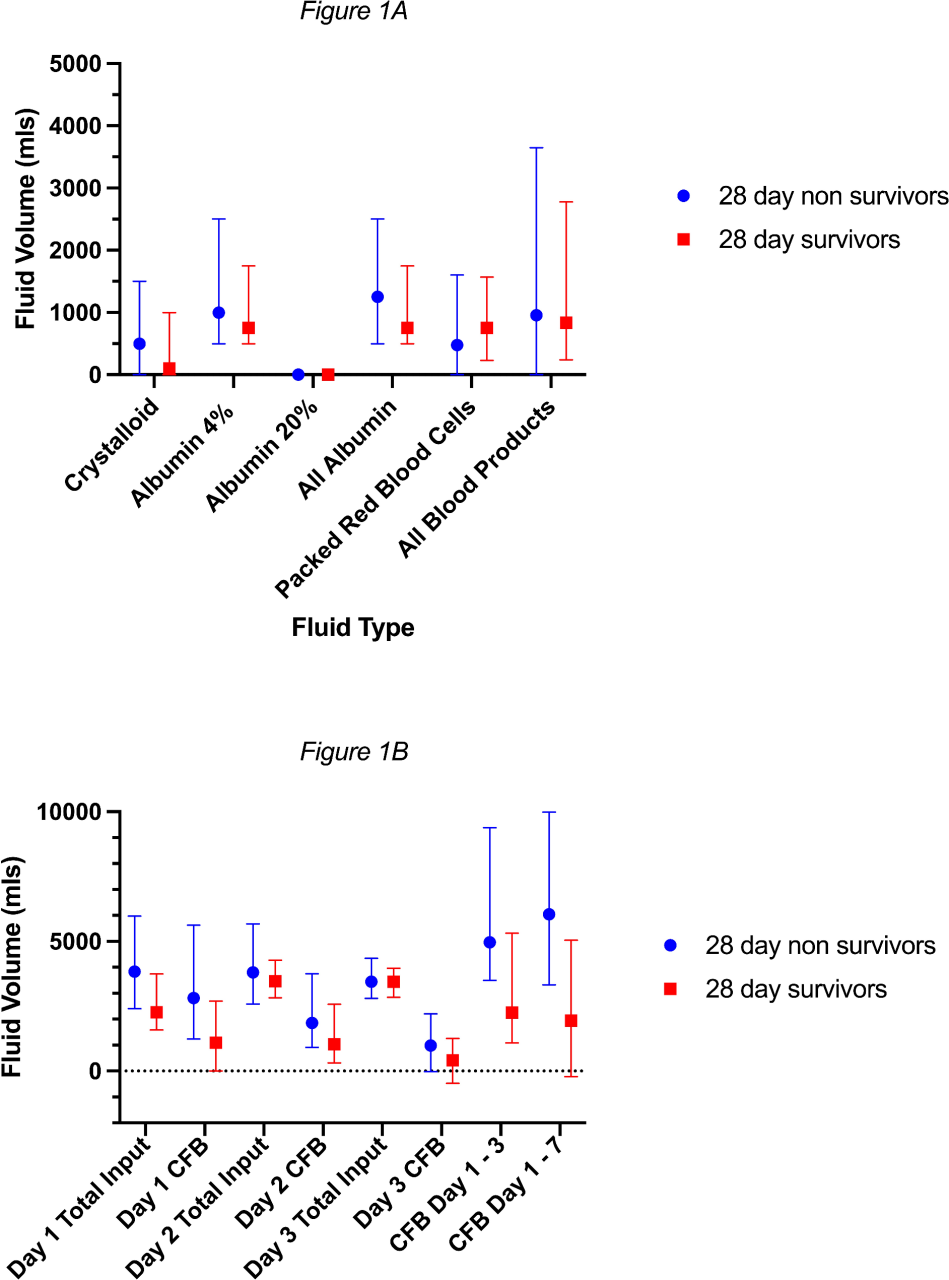
**A**. Displaying differences in total fluid input per day from days 1 to 3, and cumulative fluid balances to the end of day 3 and day 7 between 28-day survivors and non-survivors. Significant differences were noted between Day 1 CFB (*p* = 0.035), CFB day 1–3 (*p* = 0.017) and CFB day 1–7 (*p* = 0.003). All other p-values were > 0.05. **B** displaying differences in total volumes of additional fluids given to 28-day survivors and non-survivors. There was a significant difference between groups for crystalloid only (*p* = 0.04), all other p-values were > 0.05

Data on the type of additional fluids administered was available for 86% (86/100) patients. Additional intravenous fluid was widely used in the management of this cohort of patients. 4% albumin was used more frequently than crystalloids. There was low use of 20% albumin. 54.7% (47/86) of all patients received additional crystalloids, 82.6% (71/86) received 4% albumin and 9.3% (8/86) 20% albumin. Blood products were used widely with 74.4% (64/86) receiving blood products and 68.6% (59/86) receiving packed red blood cells. The median volume of crystalloids infused to day 7 (500 vs. 100 ml) was greater in 28 day non survivors (*p* = 0.04). The volumes of different types of additional fluid administered are shown in Fig. 1A (and table S1).

### Fluid balance

Table 4 shows the unadjusted and adjusted odds ratios (OR) for 28-day mortality. Each additional litre of CFB at the end of day 7 was associated with an increased 28-day mortality with an adjusted OR of 1.11 (95% CI 1.001–1.23). By the end of day 3 the adjusted OR for was 1.09 (95% CI 0.97–1.22). A sensitivity analysis excluding early deaths (< 48 h) confirmed these results.

**Table 4:** Showing univariate and multivariate logistic regression analysis of cumulative fluid balances at day 3 and 7 on the risk of 28-day mortality

	Unadjusted OR (95% CI)	Adjusted OR (95% CI) ^a^	*p* – value ^b^
Risk of 28-day mortality per 1 L increase in CFB at end of Day 3	1.14 (1.02–1.26)	1.09 (0.97–1.22)	0.152
Risk of 28-day mortality per 1 L increase in CFB at end of Day 7	1.16 (1.05–1.28)	1.11 (1.001–1.23)	0.047
**Sensitivity Analysis (excluding 19 early deaths)** ^**c**^			
Risk of 28-day mortality per 1 L increase in CFB at the end of day 3	-	1.08 (0.97–1.21)	0.157
Risk of 28-day mortality per 1 L increase in CFB at the end of day 7	-	1.11 (1.01–1.22)	0.036

OR = odd ratio, ci = confidence interval, cfb = cumulative fluid balance

^a^ Adjusted for location of cardiac arrest (OHCA vs. IHCA), ROSC and duration of ECMO

^b^ p- values are for adjusted OR

^c^ Early deaths were defined as < 48 h from ECMO initiation. This was adjusted for location of cardiac arrest and ROSC only

The daily fluid balances and inputs for day 1,2 and 3 alongside the CFB at day 3 and 7, are shown in Fig. 1B (and table S2). Median CFB for days 1 to 3 and days 1 to 7 were different between survivors and non survivors. There was a significant difference in 28-day survival (see Figure S1, S2 and Table S3) when dividing cumulative fluid balance from days 1–3 and days 1–7 into quartiles.

### OHCA vs. IHCA

**Fig. 2:**
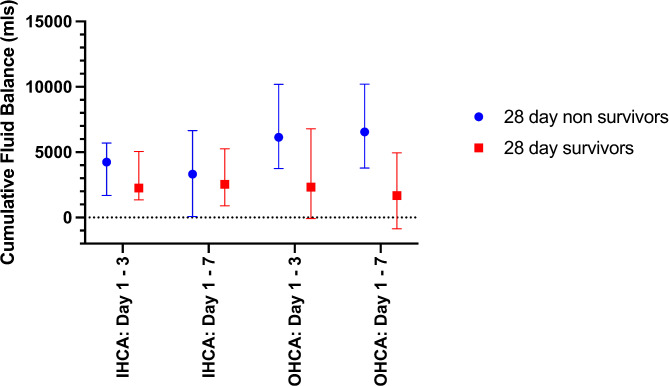
Displaying differences in median cumulative fluid balances at the end of day 3 and day 7 between 28-day survivors and non-survivors of both IHCA and OHCA. Differences between for IHCA at day 3 (*p* = 0.53) or day 7 (*p* = 0.55) were not significant. Differences for OHCA at day 3 (*p* = 0.007) and day 7 (*p* < 0.001) were significant

When location of cardiac arrest was considered, there was a significant difference in the CFB at day 3 and day 7 between 28-day survivors and non survivors in OHCA. This was not shown for IHCA patients. This is shown in Fig. 2.

## Discussion

In this retrospective analysis of ECPR patients at a high volume ECMO centre additional fluids, including 4% albumin and blood products, were widely used for patients requiring ECPR. After adjusting for location of arrest, duration of ECMO and whether there was an episode of ROSC, CFB at the end of day 3 was not significantly associated with 28-day mortality. However, by the end of day 7, a positive CFB was associated with 28-day mortality, with every 1 L increase in CFB at the end of day 7 increasing the risk by 11% (OR 1.11, 95% CI 0.1–23%).

Fluid overload has been shown to be harmful across a wide range of critically ill patients [3, 12, 13]. This includes those receiving both venovenous (VV) and venoarterial (VA) ECMO [14, 15]. An Extracorporeal Life Support Organisation consensus statement suggests aiming for a negative fluid balance for patients on all modalities of ECMO to minimise the risk of becoming fluid overloaded [4]. However, there are no randomised controlled trials assessing the effect of different fluid balance strategies, and there is very limited literature directly assessing fluid use in those requiring ECPR as a separate cohort to all-comers requiring VA-ECMO. Dong et al. [16], describes that the CFB from days 1 to 4 was significantly associated with survival to ICU discharge in both unadjusted and adjusted analyses in 72 ECPR patients whose ECMO duration was greater than 72 h. The median daily balances were all negative except for non-survivors on day 1. This differs from our cohort of patients in which the median daily fluid balances were all positive for survivors and non-survivors on days 1,2 and 3. The exclusion of those requiring ECMO for less than 72 h may result in a large proportion of those patients with profound early shocked states and unsupportable circulations not being included. This may mean the overall cohort within this study were more haemodynamically stable, allowing for more aggressive control of fluid balance. Whilst the overall use of CRRT was remarkably similar between the two studies (64% in our cohort vs. 66% by Dong et al.), it is plausible that CRRT was being used to aggressively control fluid balance.

Taira et al. conducted a secondary analysis of the SAVE-J II study [17]. This included 959 patients and only assessed the first 24 h of ICU admission following OHCA. Patients in this study were older, had higher rates of shockable initial rhythm and there were lower rates of bystander CPR. The authors found that increasing 24-hour fluid balance was not only associated with in-hospital mortality, but also unfavourable neurological outcome, AKI and need for CRRT. The results of both these studies reinforce that increasingly positive early CFB are associated with worse outcomes. This is consistent with multiple other studies assessing fluid balance in VA ECMO patients [7, 15, 18].

ECMO is associated with increased vascular permeability from immune system activation and a “pan-endothelial injury” [19, 20]. Post cardiac arrest syndrome is a complex constellation of pathophysiological processes including ischaemia – reperfusion injury, activation of inflammatory cascades and organ dysfunction leading to high rates of post resuscitation shock [21]. The combination of both these may result in different fluid requirements to those requiring VA ECMO but without cardiac arrest. Additionally, hypovolaemia can be problematic for patients on ECMO, as it may result in ECMO blood access insufficiency, and guidelines recommend addressing this with fluids or transfusion [22]. Despite these recommendations there is no robust evidence on fluid responsiveness assessments for patients on VA-ECMO making it increasingly difficult to know whether a patients will respond to fluid bolus [23]. All these factors may contribute to the liberal use of fluid following ECPR resulting in multi-organ dysfunction from fluid overload [24]. This could be compounded by the multi-faceted inflammatory response following cardiac arrest and initiation of ECMO.

Those who had an OHCA and had died by day 28 had higher median fluid balances by the end of day 3 and day 7 compared to survivors. This was not shown for those who had an IHCA. The reasons for this are likely to be multifactorial. Importantly, causes of cardiac arrest may be different [25]. Secondly, low flow times were shorter in the IHCA group (45 vs. 60 min). Shorter low flow times may result in less of an inflammatory response which may in turn impact the fluid requirements post cardiac arrest. Modulation of this inflammatory response may be important [26].

There is no literature assessing the type of fluid administered to patients both post cardiac arrest, and more specifically those requiring ECPR. The American Heart Association and Neurocritical Care Society Guidelines acknowledge the lack of literature comparing differing fluid strategies [27] and suggest balancing the risk between cerebral oedema and risks of hyperchloraemia when choosing intravenous fluid post cardiac arrest. Within this study albumin was widely used. Wengenmeyer et al. [28] found that albumin administration was independently associated with hospital survival for those on VA ECMO. In this study, approximately two thirds of the 283-patient cohort required ECPR. This difference in survival was not seemingly explained by differences in total volume of fluid administered to patients so it is plausible that albumin has other protective properties for patients requiring VA ECMO. Alongside albumin administration, blood products were also frequently used. Additional to the bleeding risks associated with ECMO and CPR associated sequalae [29], patients requiring extracorporeal cardiac support demonstrate several haemostatic derangements [30] that may necessitate transfusion. Transfusion of blood products has important implications including cost and transfusion associated harm. An association between blood product transfusion and mortality has been demonstrated [31], with VA ECMO patients having higher transfusion requirements than VV ECMO patients [32]. Better understanding of transfusion requirements in the ECPR cohort has important resource implications.

### Strengths and limitations

This study is one of the first to report the association of increasing cumulative fluid balance and mortality in ECPR patients and to our knowledge, is the first to describe the pattern of early fluid administration in this cohort of patients. Clear definitions were used, and outcome data came from a prospectively collected registry, utilising trained data collectors, with quality control and benchmarking. Given the median duration of support was two days for those who died, the inclusion of patients requiring all durations of ECPR enhances the robustness of these findings.

This study also has several limitations. Firstly, this is a retrospective study and the interplay between fluid management and mortality complex, therefore unknown confounding may have impacted the results. Secondly, data was from a single centre, which reduces the generalisability of the findings. Thirdly, despite this being the largest cohort assessing CFB beyond 24 h in ECPR patients, the sample size is still relatively small and limits the number of variables we could include in our model. Therefore, clinically important variables may have been missed. Fourth, in a small proportion of patients that were transferred from other hospitals, we were unable to locate some of their fluid management data. Fifth, data about fluid administration prior to ECMO initiation was not collected. Sixth, we defined each day as a 24-hour period commencing at 00:00; therefore, the fluid balances recorded on day one may not be over a 24-hour period. Seventh, we did not collect data on specific CRRT variables, such as ultrafiltration (UF) rates; and whilst rates of CRRT were similar between groups there may have been a difference in UF received between non-survivors and survivors. Finally, although this study only includes patients requiring ECPR, this is still a heterogenous population both with respect to the cause of the cardiac arrest and the location of arrest (in-hospital versus out-of-hospital) so there may be subgroups that are yet to be identified that have different requirements.

The impact of restrictive fluid management strategies, including assessment of optimal UF rates [33], in those requiring ECPR should be assessed in prospective trials to inform further practice. The effect in different populations (IHCA vs. OHCA) should also be evaluated.

## Conclusion

A one litre increment in CFB at the end of day 3 was not associated with 28-day mortality. By the end of day 7 a one litre increment in CFB was associated with an 11% increase in day 28 mortality. Prospective trials assessing the impact of restrictive fluid management strategies in those requiring ECPR should be conducted.

## Electronic supplementary material

Below is the link to the electronic supplementary material.

Supplementary Material 1

## Data Availability

The dataset used is available from the authors on reasonable request with appropriate approval.

## Abbreviations

CFB: Cumulative Fluid Balance
CPR: Cardiopulmonary Resuscitation
CRRT: Continuous Renal Replacement Therapy
ECMO: Extra-corporeal Membrane Oxygenation
ECPR: Extra-corporeal Cardiopulmonary Resuscitation
ICU: Intensive Care Unit
IHCA: In-Hospital Cardiac Arrest
IQR: Interquartile Range
OHCA: Out-of-Hospital Cardiac Arrest
OR: Odds Ratio
SD: Standard Deviation
UF: Ultrafiltration
VA: Veno-Arterial
VV: Veno-Venous
