# Supplementary material for: Early fluid balance and mortality following extracorporeal cardiopulmonary resuscitation: a high volume, single center study
Source: Scand J Trauma Resusc Emerg Med. 2025 Apr 22;33:66. doi: 10.1186/s13049-025-01381-8 (PMC12016082; doi:10.1186/s13049-025-01381-8)
Supplement: Supplementary file 1 — Supplementary Material 1 [file 13049_2025_1381_MOESM1_ESM.pdf]

Online Data Supplement for Early Fluid Balance and Mortality following Extracorporeal Cardiopulmonary Resuscitation: A High Volume, Single Center, Study

Humphrey GM Walker [1,2,3] , Alexander S Richardson [1,4], Arne Diehl [1,4], Brooke Riley [1], Eldho Paul [4], Aidan Burrell[1,4]

1. Department of Intensive Care and Hyperbaric Medicine, The Alfred, Melbourne, VIC, Australia
2. Department of Critical Care, St Vincent's Hospital, Melbourne, VIC, Australia
3. University of Melbourne, Department of Critical Care, Melbourne, VIC, Australia
4. Australian and New Zealand Intensive Care Research Centre, School of Public Health and Preventive Medicine, Monash University, Melbourne, VIC, Australia

Contents

Data Dictionary

Table S1 showing total volume of additional fluid prescribed to death, day 7 or ECMO decannulation

Table S2 showing total fluid input, daily cumulative fluid balances for days 1,2 and 3 and cumulative fluid balances from day 1 to 3, and day 1 to 7

Table S3 showing cumulative fluid balances (CFB) per quartile

Figure S1 showing Kaplan-Meier curve depicting probability of 28-day mortality according to CFB quartiles from ECMO initiation to the end of day 3.

Figure S2 showing Kaplan-Meier curve depicting probability of 28-day mortality according CFB quartiles from ECMO initiation to the end of day 7.

Data Dictionary

In addition to the definitions given in the manuscript:

- Location of Arrest: The location at which the initial arrest occurred.
- Comorbidities: Any history of the documented co-morbidities in any of the EMR documentation
- Witnessed Arrest: someone present at time of collapse.
- Bystander CPR: Any CPR prior to emergency service arrival. This may not have been immediately following cardiac arrest.
- Shockable: Ventricular Fibrillation / Ventricular Tachycardia as first recorded rhythm
- No Flow Time: Time from arrest to commencement of CPR
- Low Flow Time: Time from commencement of CPR until establishment of ECMO flows
- ROSC (Return of Spontaneous Circulation): Any documented episode prior to establishment ECMO flows. No temporal requirement was needed.
- Bedside cannulation location included in ICU, ED or on the ward.
- LV Decompression included use of surgical LV vent, IABP, Impella
- CRRT on ECMO: any documented CRRT whilst on ECMO within first 7 days
- High dose vasopressors: a noradrenaline dose > 0.26mcg/kg/min or addition of vasopressin at any time in first 3 days. This is based on Surviving Sepsis Guidelines [1]
- Bleeding: Defined as clinically significant and/or associated with the need for > 3 unit of packed red blood cells in a 24-hour period, a drop in haemoglobin of > 2g/dL or a surgical, radiological or other interventional procedure to control or investigate the bleeding
- Crystalloid fluid included 0.9% sodium chloride and Hartmann's solution.
- Blood products included packed red blood cells, platelets, fresh frozen plasma, and cryoprecipitate.
- Fluid Input: Sum of all administered fluids
- Daily fluid balance: Daily output subtracted from daily input.
- ECMO survival: Successful liberation from ECMO without arrest in the operating theatre
- Hospital Survival: Discharged from Alfred Health
- Brain Death: formal documented brain death examination was required.
- Withdrawal of Life Sustaining Therapies – Neurological: All causes where neurological impairment or injury was primary cause of death and patient was not formally brain dead.
- Withdrawal of Life Sustaining Therapies – Non-Neurological: All causes where no evidence that patient had clearly sustained a significant neurological injury but life sustaining therapies withdrawn. Unsupportable circulation was a subset of this.
    - Unsupportable Circulation: Escalating vasopressors listed as reason as to why support was being withdrawn.
- Organ Donation: Organ retrieval in operating theatre

Table S1 showing total volume of additional fluid prescribed to death, day 7 or ECMO decannulation

	Number of patients receiving, n (%)	Entire Cohort (n = 86)	Dead at day 28 (n = 41)	Alive at day 28 (n = 45)	p – value
Crystalloid, mls	47 (54.7)	250 (0 – 1000)	500 (0 – 1500)	100 (0 – 1000)	0.04
4% Albumin, mls	71 (82.6)	1000 (500 – 2000)	1000 (500 – 2500)	750 (500 - 1750)	0.12
20% albumin, mls	8 (9.3) [a]	0 (0 – 0)	0 (0 - 0)	0 (0 - 0)	0.70
All Blood Products, mls	64 (74.4)	939 (0 – 3382)	955 (0 - 3650)	834 (240 – 2778)	0.45
Packed Red Blood Cells, mls	59 (68.6)	548 (0 – 1603)	478 (0 – 1603)	750 (230 – 1568)	0.70

All values are median and IQR. The number of patients differ due to not all patients included in the study having balances available for types of fluid received. Volumes were censored at death, decannulation or at the end of day 7 if remaining on ECMO. The number of patients receiving reflects the number of patients (out of 86) receiving any volume of given fluid.

[a] 7 of these received 4% albumin, resulting in 72 (83.7%) patients receiving either 4% or 20% albumin

Table S2 showing total fluid input, daily cumulative fluid balances for days 1,2 and 3 and cumulative fluid balances from day 1 to 3, and day 1 to 7				
	**Entire Cohort**	**Died by day 28**	**Survived to day 28**	**p - value**
**Day 1 (ml)**				
Total Fluid Input	3104 (1915 – 5432)	3838 (2402 – 5979)	2263 (1585 – 3745)	0.062
Daily Fluid Balance	1806 (530 – 3703)	2814 (1235 – 5622)	1095 (0 – 2695)	0.035
**Day 2 (ml)**				
Total Fluid Input	3620 (2811 – 5171)	3807 (2583 – 5670)	3461 (2825 – 4275)	0.183
Daily Fluid Balance	1495 (510 – 2960)	1850 (912 – 3751)	1032 (319 – 2572)	0.171
**Day 3 (ml)**				
Total Fluid Input	3442 (2806 – 4009)	3442 (2805 – 4346)	3448 (2845 – 3961)	0.356
Daily Fluid Balance	723 (-180 – 1408)	983 (-28 – 2209)	412 (-478 – 1250)	0.025
**Cumulative Fluid Balances**				
Day 1 – 3	4067 (1559 – 7558)	4969 (3491 – 9387)	2258 (1086 – 5319)	0.017
Day 1 - 7	3673 (1269 – 7174)	6044 (3321 – 9989)	1947 (-219 – 5040)	0.003

**Table S3 showing cumulative fluid balances (CFB) per quartile**		
	**CFB to the end of day 3 (L)**	**CFB to the end of day 7 (L)**
Quartile 1	-11.8 to 1.5	-18.1 to 1.2
Quartile 2	1.5 to 4.0	1.3 to 3.6
Quartile 3	4.0 to 7.6	3.6 to 6.8
Quartile 4	7.6 to 15.6	6.8 to 19.9

Volumes were censored at death, decannulation or at the end of day 7 if remaining on ECMO. Quartiles range from lowest cumulative fluid balances (Q1) to the highest cumulative fluid balances (Q4)

Figure S1 showing Kaplan-Meier curve depicting probability of 28-day mortality according to CFB quartiles from ECMO initiation to the end of day 3.

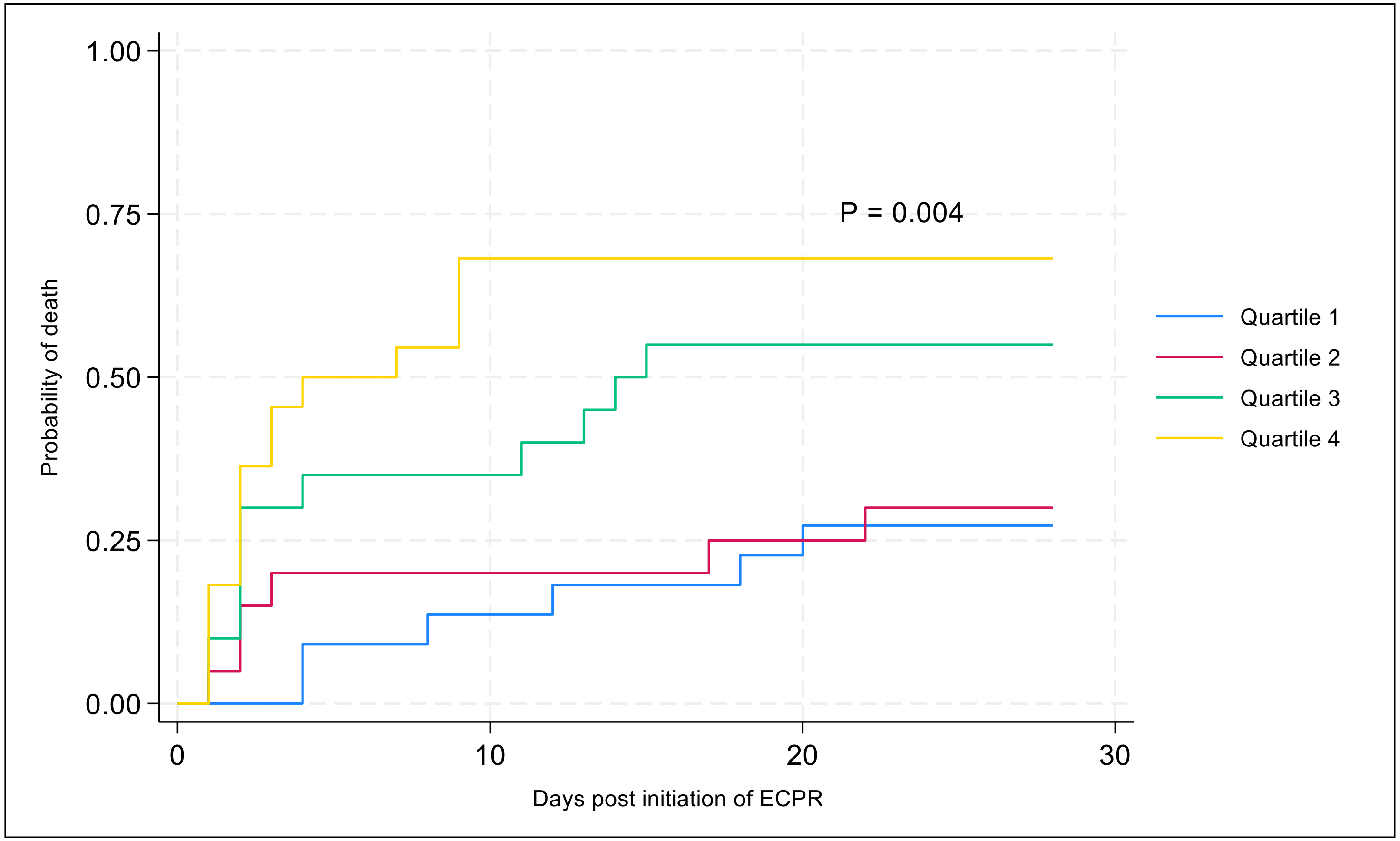

The smallest cumulative balance is depicted by quartile 1, and the largest by quartile 4. The values for the quartiles are given in Table S1.

Figure S2 showing Kaplan-Meier curve depicting probability of 28-day mortality according CFB quartiles from ECMO initiation to the end of day 7.

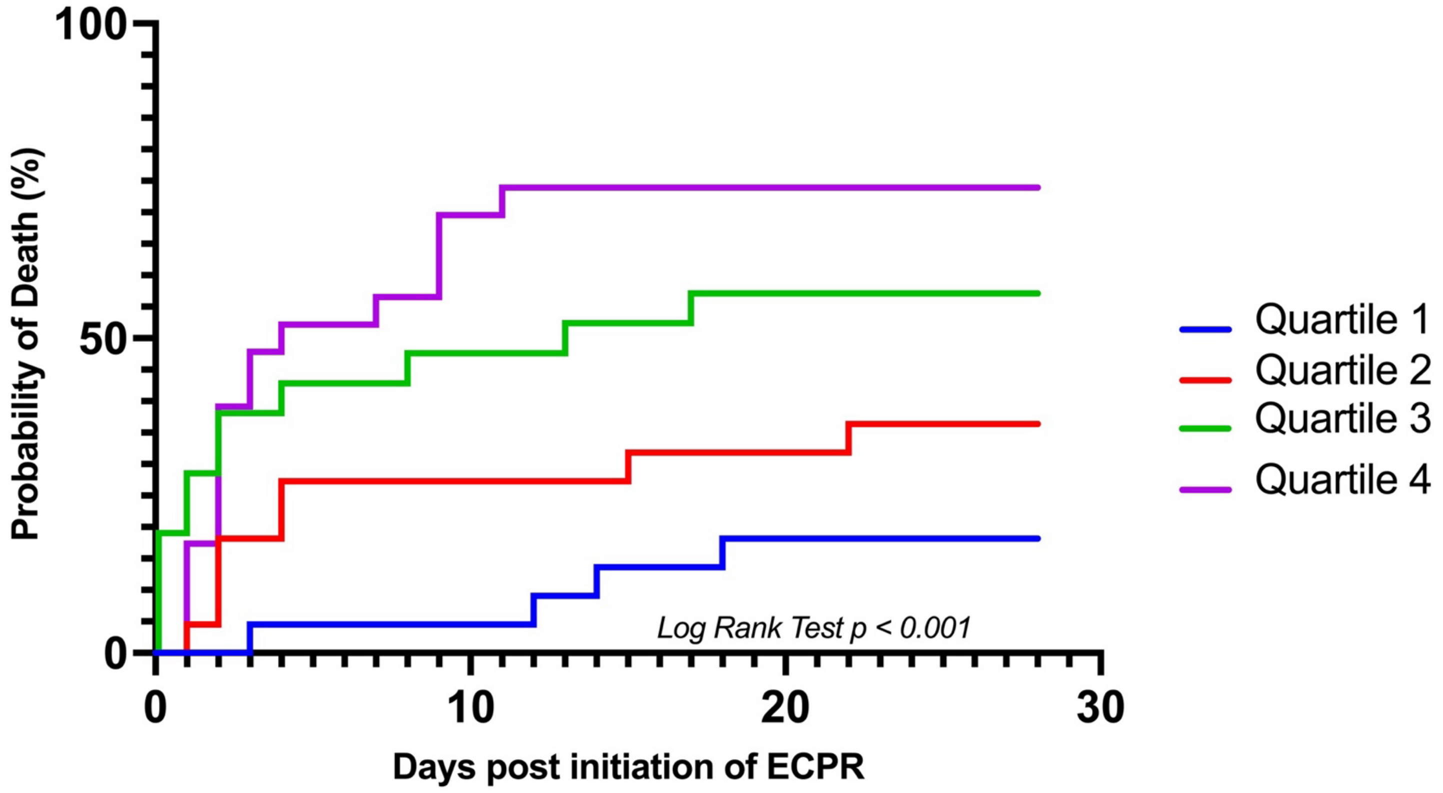

The smallest cumulative balance is depicted by quartile 1, and the largest by quartile 4. The values for the quartiles are given in Table S1
